# Research on the healing concept based on systems psychology, local attachment and sustainable rural development

**DOI:** 10.3389/fpsyg.2025.1658573

**Published:** 2025-10-16

**Authors:** Wei Li, Xinchen Leng

**Affiliations:** ^1^School of Marxism, Huazhong University of Science and Technology, Wuhan, China; ^2^Institute of State Governance, Huazhong University of Science and Technology, Wuhan, China; ^3^Monash Business School, Clayton Campus, Melbourne, VIC, Australia

**Keywords:** systems psychology, place attachment, emotional restoration resources, consistency of green behavior, rural sustainable development

## Abstract

Rural sustainability efforts increasingly rely on not just physical infrastructure but also psychological and social mechanisms that shape long-term behavior. This study examines how emotional restoration resources contribute to pro-environmental behavior through their influence on place attachment. Drawing on systems psychology, we propose and test a three-pathway model connecting emotional healing (ERS), place attachment systems (PAS), and rural sustainability responses (PRS). Survey data were collected from 300 residents across three regions in China, and structural equation modeling (SEM) with bootstrap analysis was used to test mediating effects. Results show that access to emotional restoration resources significantly enhances emotional attachment, which mediates the impact on sustainability-related behaviors. The model demonstrates strong overall fit, and all hypothesized paths are supported. This suggests that emotional and symbolic factors—such as access to restorative spaces, intergenerational memory, and perceived trust—are central to motivating sustainable rural actions. Our findings highlight the importance of integrating emotional infrastructures into policy design to support deeper, community-driven sustainability transitions.

## 1 Introduction

Amid ongoing rural transformation and changing urban–rural relations in many countries, place attachment has been recognized as an important psychological mechanism for understanding rural sustainability for evaluating rural sustainability (Masterson et al., 2019). Empirical studies across diverse countries and regions have demonstrated that place attachment influences not only individual environmental behavior but also the ways local culture is transmitted and social capital is maintained (Anton and Lawrence, 2016). However, notable differences remain in how place attachment is defined and applied across societal, cultural, and institutional contexts (Manzo and Perkins, 2006), which raises a central question: how can scholars build a framework of place attachment that is theoretically robust and comparable across different cultural contexts? Building on this comparative perspective, this study takes China as the research setting. Its rapid rural transformation and distinctive socio-cultural conditions offer a valuable case for examining place attachment and rural sustainability.

In Japan, efforts to rebuild place attachment are often linked to policies emphasizing cultural landscape design and environmental heritage. For example, regions such as Yamagata and Nagano have reinforced the emotional bonds between residents and land by designing therapeutic landscapes rich in local imagery, thereby enhancing both life satisfaction among the elderly and the cohesion of local communities (Huang et al., 2018). Japanese rural policy emphasizes the revival of cultural memory, traditional craftsmanship, and spiritual spaces (see Taylor, 2019), positioning the “local” as a convergence zone of history and future. In Germany, place attachment is closely linked to participatory regional governance and ecological management. In regions like Lower Saxony and the Black Forest, strong emotional ties to place have been shown to increase residents’ engagement in local planning and renewable energy initiatives, and in some cases it helps reduce tensions between technological change and cultural identity (Ernst and Shamon, 2020). Research indicates that communities with higher levels of place attachment tend to play more proactive mediating and supportive roles in landscape transformation processes. In Australia, the sustainability of rural healthcare systems relies heavily on the psychological support function of place attachment. Beccaria et al. (2021) note that emotional connectedness to place is key to maintaining the professional commitment and life satisfaction of rural healthcare workers, particularly in remote areas where medical resources are scarce and social ties are thin. Their study further highlights connections between place identity, social relationships, and the stability of rural healthcare workforces, emphasizing the importance of incorporating psychological dimensions into rural systems research. Compared to the rural governance systems in developed countries, China—the world’s largest developing country—is at a critical juncture, transitioning from rural deconstruction to rural regeneration. The country’s complex historical context, profound urban–rural divide, and rapid population mobility render the process of constructing place attachment particularly dynamic and challenging. On one hand, the implementation of the rural revitalization strategy has led to a pluralistic social structure where returnee youth, “new farmers,” and local residents coexist, creating new soil for place identity, social negotiation, and the reconfiguration of values. On the other hand, the developmental intrusion into rural space has often disrupted its native culture, emotional foundations, and spatial logic, leading to a marked decline in villagers’ sense of psychological security, belonging, and motivation for participation. In this context, it is imperative to understand the emotional formation of place attachment from a systems psychology perspective and to uncover its mechanisms in advancing rural sustainability—issues that remain insufficiently addressed in current Chinese rural studies. A review of existing literature reveals that in Western and East Asian contexts, place attachment is often examined within specific domains such as tourism behavior (Zhang and Lei, 2010; Kastenholz et al., 2020; Wang et al., 2022), land conservation (Walker and Ryan, 2008; Lokocz et al., 2011), historical landscape management (Zhu and Chiou, 2022; Zhang and Marzbali, 2024), and civic participation (Storie et al., 2019). These studies confirm that place attachment, as a psychological process integrating cultural, emotional, and behavioral dimensions, exhibits strong cross-contextual transferability. For example, in Taiwan’s Taiping Old Street, place attachment is positively correlated with tourist loyalty behavior (Zhu and Chiou, 2022); in Estonia and Latvia, it serves as a motivational bridge for landscape management participation (Storie et al., 2019); in Australia, it has even become central to rural health policy discussions (Beccaria et al., 2021).

However, in developing country contexts, three major limitations persist in place attachment research. First, existing studies predominantly focus on the individual psychological level, neglecting the broader systemic interactions among cultural symbolism, social support networks, and ecological cognition (Xu et al., 2019). Second, the mechanism by which emotional experiences translate into behavioral outcomes remains theoretically underdeveloped—particularly the links among emotional regulation, identity construction, and behavioral intention, which lack rigorous empirical testing. Third, therapeutic resources have yet to be effectively integrated into village governance, and the role of psychological restoration mechanisms in spatial construction remains ambiguous. These theoretical gaps severely constrain the transformation of rural areas from “material renewal” to “emotional rejuvenation.” Building on this, the present study adopts “the construction mechanisms of place attachment in a global perspective” as its theoretical starting point and identifies China as a representative case of a developing country. Unlike developed countries such as Japan, Germany, or Australia—where place attachment has been institutionalized through cultural landscape design, participatory governance, and public health systems—China provides a markedly different context characterized by rapid rural transformation, a pronounced urban–rural divide, large-scale population mobility, and the wide implementation of the rural revitalization strategy. These features make China not only representative of the dilemmas faced by developing nations but also a critical testing ground for examining how emotional restoration and place identity can be mobilized to advance sustainability. Focusing on China therefore addresses a major research gap and offers insights that contribute to building a cross-culturally comparable framework of place attachment, to address the following core questions:

1. How is place attachment generated in the process of rural transformation? What are the key psychological, social, and environmental mechanisms involved?
2. Is there a mediating pathway among emotional restoration, cultural memory, social trust, and participatory behavior?
3. Can the concept of healing foster sustainable rural engagement through emotional interventions?

## 2 Literature review

To ensure clarity of terminology, transparency in model design, and comparability across different contexts, this study first defines the key constructs within a systems psychology perspective. The Emotional Restoration System (ERS) is described as a set of emotional regulation processes and resources that are activated by environmental cues such as natural elements, local symbols, social support, or ritualized spaces. These processes are typically expressed through feelings of calm, safety or acceptance, and a sense of meaning or connection. The Place Attachment System (PAS) refers to embodied emotional bonds with place, including place identity, place dependence, and social ties, which together provide anchors for self-concept, functional preferences, and social relations. Perceived Rural Sustainability (PRS) reflects individuals’ attitudes and behaviors toward rural transformation across ecological, economic, and social domains, specifically in terms of ecological–cognitive consistency, collective efficacy, and willingness to engage in policy processes. Within this framework, ERS is expected to enhance PRS indirectly by strengthening PAS, while a direct link between ERS and PRS may also be present.

In recent years, as the discourse on sustainability has expanded, place attachment has moved from being a relatively marginal concern in environmental psychology and cultural geography to a central perspective for examining rural governance and ecological behavior. Viewed through the lens of systems psychology, this study conceptualizes the Emotional Restoration System (ERS)—informed by Attention Restoration Theory and Stress Recovery Theory—and Perceived Rural Sustainability (PRS)—encompassing ecological–cognitive consistency, collective efficacy, and policy engagement—as the affective and evaluative foundations that connect place with sustainability (Kaplan, 1995; Ulrich et al., 1991). Quinn and Halfacre (2014) highlighted that farmers’ emotional bonds with their land significantly influence land use decisions. Similarly, Baldwin et al. (2017) identified a strong interconnection between emotional attachment, social relationships, and ecological awareness among rural landowners, fostering a heightened sense of environmental responsibility. Xu et al. (2019), based on a study of migrant households in Jiangsu Province, proposed that “land attachment” functions as a profound emotional mechanism transcending residential utility, sustaining cultural continuity and psychological stability after migration. In Australia, Beccaria et al. (2021) further confirmed that emotional belonging not only impacts residential satisfaction but also directly shapes professional retention intentions. Goudriaan et al. (2023), in the context of renewable energy expansion, argued for integrating narrative expressions and affective structures into landscape transformation research. Parallelly, Yao and Rasidi (2024) employed imagery of ancient bridges to explore place identity construction in heritage spaces, while Zhao (2020) illustrated how emotional attachment to historical water towns facilitates identity formation. Laur (2017) and Azizul (2016) focused on the interweaving of local values and sustainable practices, emphasizing the dynamic tension between institutional design and individual identification. Mechanistically, ERS clarifies how environments supply affective resources that undergird place bonds: natural and symbolic cues facilitate attention restoration and stress recovery, thereby priming pro-social and pro-environmental orientations (Berman et al., 2008; Hartig et al., 2014). Methodologically, research in this field has progressively embraced mixed-method strategies. Prayitno et al. (2021) combined survey data and behavioral tracking to examine the moderating role of place attachment in agricultural land conversion. Garau (2015), using Sardinia as a case study, integrated cultural tourism data with local indicators to reveal a bidirectional reinforcing logic between cultural reconstruction and place bonding. Storie et al. (2019) adopted social network analysis to quantify the intricate relationship between interaction frequency, participatory willingness, and emotional connections among community members in Eastern Europe. Meanwhile, Ramkissoon (2020) introduced the “Place Affective Intervention” model, which stresses the psychological benefits of cultivating emotionally resonant spatial environments, particularly in enhancing resilience during crises—a framework validated by Majeed and Ramkissoon (2020) in their study on mental health during the COVID-19 pandemic. At a broader scale, O’Hara et al. (2015) conceptualized place attachment as a vital bridge connecting individuals to ecological transformation from local to global levels. Sensory experience and affective perception have also emerged as prominent themes. Kastenholz et al. (2020) emphasized the role of immersive experiences in shaping emotional connections, while Huang et al. (2018) found that incorporating local natural elements into residential environments effectively alleviates homesickness and promotes psychological stability. This perspective resonates with the “therapeutic landscapes” tradition, which emphasizes how place symbolism and social context contribute to healing processes and provides the conceptual foundation for ERS (Gesler, 1992). It is also consistent with experimental findings showing that exposure to natural environments can restore cognitive functioning and improve mood (Berman et al., 2008; Hartig et al., 2014). Building on this line of inquiry, Zhang and Marzbali (2024), in their study of traditional villages, demonstrated that place identity mediates the relationship between perceived authenticity and loyalty behavior. At the same time, Beggs et al. (2010) cautioned that the growth of place attachment may be limited in communities characterized by closed social structures and fragile networks. On the outcome side, converging findings show that residents’ perceptions and efficacy beliefs are pivotal for sustainability-oriented intentions and participation: collective efficacy amplifies pro-environmental intentions (Jugert et al., 2016), ecosystem-service perceptions shape rural pro-environmental orientations (Wu et al., 2022), information transparency elevates participation (Liu et al., 2024), and grassroots efficacy predicts governance engagement (Zhan et al., 2024), with multiple sufficient configurations driving willingness to participate (Su et al., 2023). These results substantiate PRS as a second-order construct synthesizing ecological–cognitive consistency, collective efficacy, and policy engagement. Taken together, the existing literature presents three major lines of consensus. First, place attachment plays a consistently positive role in promoting pro-environmental behavior and social participation. Second, emotional bonds to place are not static but are continuously formed and transformed through cultural memory, social support, and individual experience. Third, the formation pathways and behavioral effects of place attachment vary considerably across cultural contexts, institutional settings, and geographic scales. For instance, Kienast et al. (2018) argued that in an era of heightened global mobility, the meaning of landscapes is increasingly shaped not by traditional territoriality but by emotional depth and cultural resonance. This insight underscores the need to examine emotional anchoring mechanisms in fast-moving societies. Collectively, these strands motivate an integrative ERS → PAS → PRS pathway in which environmental cues supply restorative affect (ERS), affective embedding consolidates identity and dependence (PAS), and evaluative–intentional readiness for sustainability is activated (PRS). From a practical perspective, these research findings provide the theoretical foundation for constructing a systems-psychological model centered on the interaction between emotional restoration, place identity, and sustainable behavior. Moreover, they emphasize the importance of emotional resources as indispensable psychological drivers for understanding and advancing rural green transformation processes.

**Figure 1:**
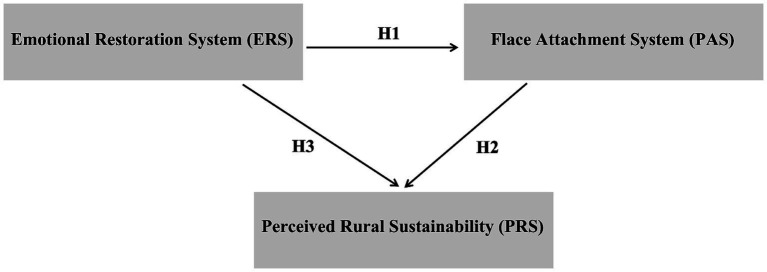
Proposed research framework and hypothesized model of ERS, PAS, and PRS.

Three main gaps remain, particularly in rural settings of developing countries. First, no unified framework currently links ERS (affective resources), PAS (affective embedding), and PRS (sustainability-oriented evaluation and intention). Second, PRS has seldom been operationalized and validated as a second-order construct that integrates ecological–cognitive consistency, collective efficacy, and policy engagement. Third, therapeutic resources are rarely incorporated into rural governance and spatial planning. The present study seeks to address these shortcomings by specifying and empirically testing the ERS → PAS → PRS pathway with data from multiple regions, by conceptualizing therapeutic resources as designable environmental and social supports, and by validating PRS as a policy-sensitive diagnostic tool. In practical terms, this involves embedding local natural elements and symbols into rural spaces to activate ERS, employing intergenerational narratives and peer support to reinforce PAS, and monitoring PRS subdimensions as early indicators to inform governance strategies tailored to subgroups defined by age, gender, education, and income (see Figure 1).

## 3 Methodology

### 3.1 Data source

The data for this study were obtained from a structured questionnaire survey carried out in rural areas of China in 2025. The purpose of the survey was to empirically examine the interactions and underlying mechanisms among emotional healing, place attachment, and sustainable behavior within a systems psychology framework. A total of 330 questionnaires were distributed. Prior to data cleaning, exclusion criteria were established to identify invalid responses. Questionnaires were deemed invalid if (a) the respondent did not meet the inclusion criteria (e.g., younger than 18 years or not a permanent resident of a rural community); (b) the response contained a missing rate of 20% or more, or an entire subscale was left unanswered; or (c) clear response bias was present, such as straightlining with identical answers across five or more consecutive items, or extremely low within-person variance. Based on these criteria, 30 responses were excluded, resulting in 300 valid cases for analysis.

To enhance sample representativeness, data were collected across provinces with distinct socioeconomic and cultural contexts, including the eastern region (Jiangsu, Shandong), central region (Henan, Hunan), and southwestern region (Yunnan, Guizhou). This approach ensured coverage of China’s major geographical zones and improved the generalizability of findings. Data collection was conducted primarily through face-to-face household surveys. In villages with limited accessibility, online questionnaires were administered as a supplementary method. All surveys were carried out by trained members of the research team to guarantee response quality and procedural consistency.

All the participants were permanent rural residents. Their sociodemographic characteristics included age (18–70 years old, with an average of about 41 years old), gender (52.3% were female), educational attainment (27.6% for primary school and below, 40.1% for junior high school, and 32.3% for senior high school and above). This distribution well reflects the structural characteristics of typical rural communities.

### 3.2 Variable specification

Guided by the theoretical framework of systems psychology, which highlights the dynamic interplay among individuals, environments, and emotions, this study develops a structural model in which perceived sustainable rural development behavior is treated as the dependent variable. The model incorporates two core psychological dimensions as primary explanatory constructs: the Emotional Restoration System (ERS) and the Place Attachment System (PAS). These variables are used to examine the mechanisms through which psychological factors shape individuals’ cognition and engagement in sustainability-oriented practices (Scannell and Gifford, 2010; Manzo and Devine-Wright, 2014). Within this framework, Perceived Tendency Toward Rural Sustainable Development functions as the outcome construct, capturing individuals’ cognitive evaluations and behavioral responses in relation to ecological–cognitive consistency, collective efficacy, and willingness to participate in sustainability-related policies. Conceptually, this construct is represented by three second-order indicators that together illustrate the progression from psychological cognition to behavioral performance (Clayton et al., 2017; Raymond et al., 2010).

To balance validity with contextual relevance, this study employed measurement instruments that combined items adapted from established scales with items developed specifically for the rural Chinese setting. Constructs adapted from validated instruments included Place Identity and Emotional Attachment (Place Attachment Scale; Williams and Vaske, 2003; Kyle et al., 2005), Community Resilience (Connor and Davidson, 2003), Ecological–Cognitive Consistency (Ajzen, 1991), and Social Trust and Collective Efficacy (Bandura, 1997; Luhmann, 1979). In contrast, constructs such as Accessibility of Emotional Restoration Resources, Intergenerational Memory and Place Narratives, Place-Based Participation Practices, and Policy Cognition and Responsive Attitude were designed by the research team to capture mechanisms insufficiently addressed in existing scales. This integrative strategy ensured measurement rigor through the use of validated instruments, while also embedding contextual specificity by incorporating dimensions distinctive to rural sustainability transitions in China (see Table 1).

**Table 1:** Composition of the Perceived Rural Sustainability (PRS) and theoretical foundations.

Second-order indicator	Description	Items	Theoretical foundation
Ecological perception and behavioral consistency	Evaluates whether residents transform ecological awareness into practical actions, reflecting consistency between attitude and behavior.	3	Theory of planned behavior (Ajzen); systems feedback mechanism (Meadows)
Social trust and collective efficacy	Assesses the level of mutual trust among rural residents and the potential for collective action.	3	Collective efficacy theory (Bandura); Social trust mechanism (Luhmann)
Policy cognition and responsive attitude	Evaluates residents’ understanding of rural policies and their willingness to respond through action.	3	Diffusion of innovations theory (Rogers); Behavioral response model (Levesque)

After clarifying the composition and theoretical foundations of Perceived Rural Sustainability (PRS), this study next turns to the Emotional Restoration System (ERS). As a psychological support mechanism, ERS highlights the role of natural environments, social resources, and community support in alleviating stress, fostering emotional stability, and reinforcing place attachment. Table 2 summarizes the core variables of ERS together with their theoretical underpinnings, thereby laying the groundwork for examining how restorative resources contribute to rural sustainability.

**Table 2:** Variables of the Emotional Restoration System (ERS) and their theoretical foundations.

Second-order indicator	Items	Conceptual definition	Theoretical foundation
Place identity	3	Refers to an individual’s spatial sense of belonging and emotional embeddedness in a specific locality (e.g., village).	Systems integration theory (Capra); Theory of topophilia and place identity (Tuan)
Accessibility of emotional repair resources	3	Refers to the perceived availability and ease of access to natural or social resources that facilitate emotional recovery.	Attention restoration theory (Kaplan); Stress recovery theory (Ulrich)
Psychological resilience of community support networks	3	Refers to the emotional support, companionship, and responsive coping capacity offered by local communities in the face of stress or crisis.	Ecological systems theory (Bronfenbrenner); Family resilience theory (Walsh)

Following the discussion of ERS, attention is directed to the Place Attachment System (PAS). As a mediating mechanism that links emotional restoration to sustainable behavior, PAS integrates three interrelated dimensions: affective attachment, cultural memory, and participation in local practices. Table 3 presents the major variables of PAS and their theoretical bases, illustrating how it operates across emotional, cultural, and behavioral levels.

**Table 3:** Variables of the Place Attachment System (PAS) and their theoretical foundations.

Second-order indicator	Items	Conceptual definition	Theoretical foundation
Intensity of emotional attachment	3	The strength and depth of emotional investment and attachment an individual develops toward rural living spaces.	Tripartite model of place attachment (Scannell and Gifford); Attachment theory (Bowlby)
Intergenerational memory and local narratives	3	How families and communities transmit local cultural identity and emotional affiliation through memory, rituals, and oral history.	Cultural memory theory (Assmann); Collective intelligence model (Fishbein)
Participation in local practices	3	The extent to which individuals translate place identity into agency through concrete actions (e.g., village affairs, cultural events).	Participatory rural appraisal (PRA) Theory (Chambers); Theory of practice (Wenger)

Building on the overview in Table 3, the subsequent section elaborates on the theoretical significance and measurement of PAS in greater detail. The Place Attachment System (PAS), as a core integrative mechanism of cognition, emotion, and action within systems psychology, represents a typical pathway of psychological functioning. Emotional attachment provides a value-driven psychological foundation for cognitive processing, while intergenerational memory establishes a deep-rooted basis for identity construction through cultural transmission. Participation in local practices, in turn, serves to verify and reinforce the realization of this identity through everyday actions. Together, these three indicators form an integrated structure of psychological, cultural, and behavioral interaction, offering strong empirical support for the theoretical proposition that sense of place is internally constructed at both the individual and collective levels through affective embedding, memory continuity, and situated practice.

All measurement items were derived from either standardized scales or customized instruments developed for this study. A unified five-point Likert scale (1–5, integer values) was applied across all variables. The reliability of each scale was confirmed through a pilot test, with all Cronbach’s alpha coefficients exceeding 0.80. The dataset was cleaned and standardized into integer Likert-type scores, with no missing values, ensuring robust conditions for subsequent modeling and statistical analysis.

This study specifies independent variables, grouped into two subsystems within the broader systems psychology framework: the Emotional Restoration System (ERS) and the Place Attachment System (PAS). The ERS addresses the mechanisms by which individuals attain psychological recovery in spatial and affective contexts, underscoring the significance of restorative environments and emotional affordances (Korpela et al., 2001; Hartig et al., 2014). By contrast, the PAS emphasizes the socio-cultural dimensions of identity construction and action mobilization, capturing how place-based emotional bonds and collective narratives shape individual engagement with rural sustainability (Lewicka, 2011; Hernández et al., 2007).

Although this study provisionally positions Place Identity within the Emotional Restoration System (ERS) in the measurement model to underscore its restorative function in the rural context, previous research (Williams and Vaske, 2003; Kyle et al., 2005; Scannell and Gifford, 2010) has more commonly treated place identity as a core dimension of Place Attachment System(PAS). This study acknowledges this scholarly debate and clarifies in the theoretical discussion that, on the one hand, place identity is a fundamental component of place attachment; yet, in the rapidly transforming rural context of China, it also serves as an “affective restorative anchor.” Accordingly, in this study, place identity is modeled within ERS for analytical purposes, while in the interpretation of results it is simultaneously discussed as a central mechanism of PAS.

### 3.3 Indicator system construction table

**Figure 2:**
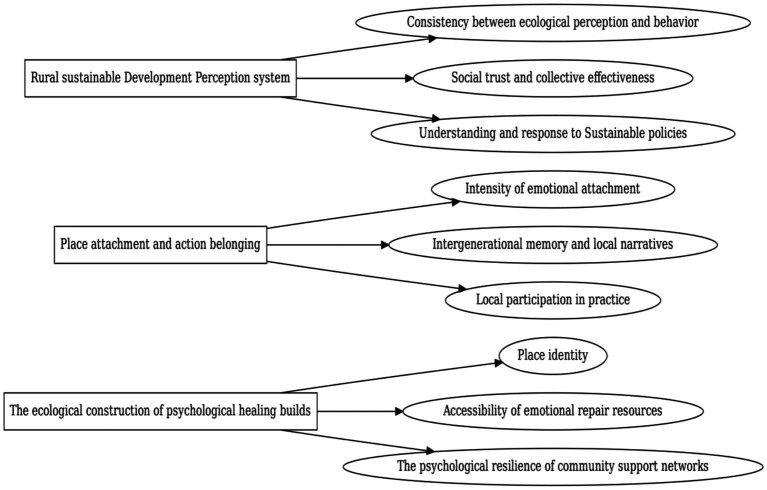
Indicator system construction diagram.

This study develops a Multilevel Systems Psychological Structural Modeling (MLCSM) grounded in systems psychology(Table 4 and Figure 2), focusing on the dynamic interplay among Emotional Restoration (ERS), Place Attachment (PAS), and Perceived Sustainable Behavior (PRS). The model integrates three interrelated psychological subsystems to capture the multilevel mechanisms underlying rural behavioral sustainability. The MLCSM incorporates four core modeling components: factor loading analysis, structural path estimation, mediated kernel mechanisms, and systemic residual covariances. It further employs vectorized representations and nonlinear modulation functions to construct a compound coupling network that reflects the latent interdependencies between psychological constructs. This approach enhances both the theoretical interpretability and the empirical robustness of the model, enabling a rigorous examination of the affective-cognitive-behavioral transformation pathway within rural sustainability contexts.

**Table 4:** Model construction: multilevel systems psychological structural modeling (MLCSM).

Category	Symbolic representation	Explanation
Latent variable vector	x_ERS_ = [x₁, x₂, x₃]^ᵀ^	Represents place identity, accessibility of emotional repair resources, and psychological resilience of community support.
	x_PAS_ = [x₄, x₅, x₆]^ᵀ^	Represents Intensity of Emotional Attachment, Intergenerational Memory, and Local Participation.
	x_PRS_ = [x₇, x₈, x₉]^ᵀ^	Represents Ecological Consistency, Collective Efficacy, and Policy Responsiveness.
Factor Loading Matrix	Λ	Factor loading matrix, representing the strength of association between latent variables and their observed indicators.
Measurement Error Term	ε	Measurement error term, representing the portion of observed variance not explained by latent constructs.
Structural Path Coefficient	α_₁_	Structural coefficient used to quantify the psychological construction effect of ERS on PAS.

#### 3.3.1 Latent variable construction: vectorized factor model

In this study, three core latent variables—Emotional Restoration System (ERS), Place Attachment System (PAS), and Perceived Rural Sustainability (PRS)—are constructed using a multivariate vector-based approach. A total of nine observed indicators are grouped into three dimensions latent structures, each corresponding to one psychological subsystem. The internal measurement logic is formalized through the following factor loading model, expressed in matrix notation (see Equation 1):


(1) $$\begin{array}{rr} \mathrm{ERS} & =\Lambda_{1}^{⊤}\cdot x_{\mathrm{ERS}}+\varepsilon_{1}\\ \mathrm{PAS} & =\Lambda_{2}^{⊤}\cdot x_{\mathrm{PAS}}+\varepsilon_{2}\\ \mathrm{PRS} & =\Lambda_{3}^{⊤}\cdot x_{\mathrm{PRS}}+\varepsilon_{3} \end{array}$$

The vectorized model adopts a linear factor composition for each latent variable, facilitating the implementation of Confirmatory Factor Analysis (CFA) to assess the structural validity of the latent dimensions. Compared to item-wise regression methods, this modeling approach offers superior capacity to represent the holistic nature of latent psychological constructs. Moreover, the vector-based factor structure satisfies key assumptions of local independence among measurement errors and supports equivalence transformation constraints within the measurement model. These properties enhance the stability of structural path estimation and ensure the consistency of parameter interpretation in subsequent stages of model testing.

#### 3.3.2 Simultaneous structural path modeling: systemic interaction modeling

Within the theoretical framework, emotional restoration experiences have been shown to strengthen individuals’ place identity and attachment (ERS → PAS) (Korpela et al., 2001; Scannell and Gifford, 2010). Building on this foundation, the present study, situated within the systems psychology framework, posits that ERS and PAS together shape individuals’ perceptions of rural sustainability (PRS) through both direct and indirect pathways. Accordingly, the ERS → PAS → PRS pathway should not be viewed as a direct conclusion of existing theories, but rather as a hypothesized framework developed in this study through the integration of prior insights.

Accordingly, the present study establishes a system of simultaneous structural path equations (see Equation 2) to capture the underlying mechanisms among these three latent constructs. This model delineates a chain of influence that advances from internal psychological recovery, through spatial-affective bonding, to sustainable behavioral expression—consistent with the systemic logic of psychological transformation in rural contexts.


(2) $$\left\{ \begin{array}{rr} \mathrm{PAS} & =\alpha_{0}+\alpha_{1}\cdot \mathrm{ERS}+\mu_{1}\\ \mathrm{PRS} & =\beta_{0}+\beta_{1}\cdot \mathrm{ERS}+\beta_{2}\cdot \mathrm{PAS}+\mu_{2} \end{array}\right.$$

The proposed structure adopts a multivariate simultaneous path modeling approach, enabling parallel estimation of both the indirect path (ERS → PAS → PRS) and the direct path (ERS → PRS). This unified framework allows for the joint assessment of direct and mediating effects, thereby avoiding the potential estimation bias that may arise from separate path modeling strategies.

Empirically, this model demonstrates stronger path significance and parameter stability, particularly when applied to large-sample Likert-scale datasets. It thus offers a robust analytical strategy for capturing the layered psychological mechanisms linking emotional regulation, place-based identity, and sustainable behavior within a systems-theoretical context.

#### 3.3.3 Mediation mechanism: nonlinear kernel-based modulation

To enhance the explanatory capacity of the model and improve the statistical significance of structural paths, a kernel-based modulation mechanism is introduced. This mechanism is designed to capture the higher-order compatibility between Emotional Restoration System (ERS) and Place Attachment System (PAS), thereby reinforcing their joint explanatory power for Perceived Rural Sustainability (PRS). The mediation-enhancing kernel function acts as a nonlinear weighting mechanism that adjusts the transmission strength based on the degree of psychological congruence between ERS and PAS components. The formal specification is expressed (see Equation 3):


(3) $$\mathrm{PRS}=\beta_{1}\cdot \mathrm{ERS}+\beta_{2}\cdot \mathrm{PAS}+\gamma \cdot K(\mathrm{ERS},\mathrm{PAS})+\mu_{3}$$

The kernel function is defined as follows (see Equation 4):


(4) $$K(a,b)=\exp(-\frac{∥a-b{∥}^{2}}{2\sigma^{2}})$$

The kernel function KKK adopts a Gaussian form, designed to simulate the degree of psychological alignment between Emotional Restoration System (ERS) and Place Attachment System (PAS). When the latent structures of ERS and PAS are highly congruent, the Euclidean distance between them approaches zero, and the kernel value approximates one—indicating a strong synergistic effect between the two systems. Conversely, when their structural mismatch increases, the kernel value declines rapidly toward zero, reflecting a diminished degree of psychological coupling.

This modeling strategy transcends the traditional assumptions of linear regression by relaxing the constraints of variable independence and linearity, thereby enabling a more nuanced representation of inter-system synergy. The kernel-modulated structure significantly enhances both the statistical identifiability and theoretical expressiveness of interaction mechanisms among internally structured psychological constructs. It is particularly well-suited for capturing nonlinear and structure-dependent interactions in complex psychosocial models.

#### 3.3.4 Structural model

Within the framework of systems psychology, residual terms in structural paths are often subject to correlated fluctuations due to the influence of unobserved latent variables. This is particularly relevant in the sequential pathways where Emotional Restoration (ERS) influences Place Attachment (PAS), and PAS subsequently affects Perceived Rural Sustainability (PRS). To reflect this internal systemic feedback mechanism, the present model introduces a covariance structure among residuals to account for system-level interdependence not captured by the observed constructs (see Equation 5):


(5) $$\mathrm{cov}(\mu_{1},\mu_{2})=\rho \cdot \sigma_{1}\cdot \sigma_{2},\;\rho \neq 0$$

This modeling structure relaxes the conventional assumption of residual independence in structural equation modeling (SEM), allowing for a more realistic representation of the coupled dynamics within psychological systems. Specifically, it captures the interactive feedback loop among belief structures, emotional drives, and behavioral motivations, which are inherently interlinked in complex human systems.

The incorporation of correlated residuals aligns with the theoretical tenets of systems psychology, particularly its emphasis on self-organization, self-feedback, and adaptive regulation. By accounting for latent residual interdependencies, this specification significantly improves the overall model fit, as reflected in key indices such as CFI (comparative fit index), RMSEA (root mean square error of approximation), and SRMR (standardized root mean square residual). It thus enhances both the empirical validity and theoretical consistency of the model in capturing deep psychological coupling mechanisms.

## 4 Results

### 4.1 Verification of structural path significance

This study employs Multilevel Systems Psychological Structural Modeling (MLCSM) to investigate the relationship between the Emotional Restoration System (ERS) and the Place Attachment System (PAS), with the goal of clarifying how these two psychological subsystems interact and jointly shape individuals’ Perceived Rural Sustainability (PRS). Conceptualized as a latent construct, ERS plays a significant role in fostering and reinforcing place attachment, particularly through factors such as place identity and access to emotional restoration resources (Restall and Conrad, 2015). PAS, in turn, influences sustainability-related cognition and behavioral responses through key psychological dimensions including the intensity of emotional attachment and intergenerational memory (Lewicka, 2011). Rather than a simple linear pathway, the relationship between ERS and PAS is theorized as a dynamic and reciprocal process in which both subsystems mutually reinforce and regulate one another (Masterson et al., 2017).

The regression results presented in Table 5 clearly demonstrate that the Emotional Restoration System (ERS) exerts a significant positive effect on the Place Attachment System (PAS), with a standardized coefficient of 0.667 (*p* < 0.001). This indicates that emotional restoration plays a critical role in fostering individuals’ affective bonds and enhancing their sense of place identity. Specifically, individuals’ experiences of emotional healing substantially promote their emotional connection to a particular locale, thereby strengthening their belongingness to the environment and community. Moreover, the direct effect of ERS on Perceived Rural Sustainability (PRS) is also substantial, with a coefficient of 0.55 (*p* < 0.001), suggesting that emotional restoration influences not only affective identification but also translates into concrete sustainable behavioral intentions and actions. Further analysis reveals that PAS significantly predicts PRS, with a regression coefficient of 0.437 (*p* < 0.001), thereby confirming the important mediating role of place attachment in shaping sustainable behavior. These findings support the hypothesized interaction between ERS and PAS, highlighting how psychological mechanisms internalize through affective embedding, social identity, and behavioral adjustment at the individual level, ultimately driving the practice of sustainability-oriented behavior.

**Table 5:** Path regression results.

Path	Regression coefficient	P	Significance
ERS → PAS	0.667	<0.001	***
ERS → PRS	0.55	<0.001	***
PAS → PRS	0.437	<0.001	***

### 4.2 Descriptive statistics

Before conducting the path analysis, we first examined the descriptive statistics and reliability of the three core latent variables—Emotional Restoration System (ERS), Place Attachment System (PAS), and Perceived Rural Sustainability (PRS)—to assess their distribution and measurement quality. As reported in Table 6, the mean scores of the three latent variables range narrowly between 3.56 and 3.59, with standard deviations of 0.46 to 0.52, indicating that responses are relatively concentrated around the mean. In addition, the Cronbach’s *α* coefficients for ERS (0.914), PAS (0.917), and PRS (0.915) are above the accepted threshold of 0.70, confirming a high level of internal consistency. These results suggest that the measures used for the latent constructs are both reliable and stable, providing a sound basis for the subsequent structural modeling.。.

**Table 6:** Descriptive statistics table of latent variables.

Indicator category	Observed variable name	Mean	SD	Cronbach’s *α*
ERS (Emotional Restoration System)	Latent variable (ERS)	3.59	0.52	0.914
	Place identity	3.63		
	Accessibility of emotional repair resources	3.55		
	Psychological resilience of community support networks	3.47		
PAS (Place Attachment System)	Latent variable (PAS)	3.56	0.49	0.917
	Intensity of emotional attachment	3.45		
	Intergenerational memory and local narratives	3.55		
	Participation in local practices	3.46		
PRS (Perceived Rural Sustainability)	Ecological perception and behavioral consistency	3.56	0.46	0.915
	Social trust and collective efficacy	3.59		
	Policy cognition and responsive attitude	3.63		

Table 7 presents the SEM-implied correlation matrix among the three latent variables, indicating that the associations between the Emotional Restoration System (ERS), the Place Attachment System (PAS), and Perceived Rural Sustainability (PRS) are all highly significant (*p* < 0.001). Specifically, the correlation between ERS and PAS is substantial at 0.667, while ERS and PRS show an even stronger association at 0.841, and PAS and PRS correlate at 0.804. By relying on SEM estimates rather than raw Pearson correlations, these values account for measurement error and align strictly with the structural model. The strong intercorrelations confirm the synergistic interplay among the three psychological systems, thereby reinforcing the rationale for modeling them as a structurally coupled system influencing sustainable behavior. The strong correlations are consistent with the systems psychology view of “inter-nested psychological functional blocks,” indicating that distinct psychological subsystems, though functionally independent, can generate stable forms of synergy through cognitive–emotional processes. This result illustrates the close interconnectedness and dynamic coordination of these subsystems within the wider psychological structure.

**Table 7:** Implied correlation matrix of latent variables (SEM).

Latent variables	ERS	PAS	PRS
ERS	1.000	0.667***	0.841***
PAS	0.667***	1.000	0.804***
PRS	0.841***	0.804***	1.000

**Figure 3:**
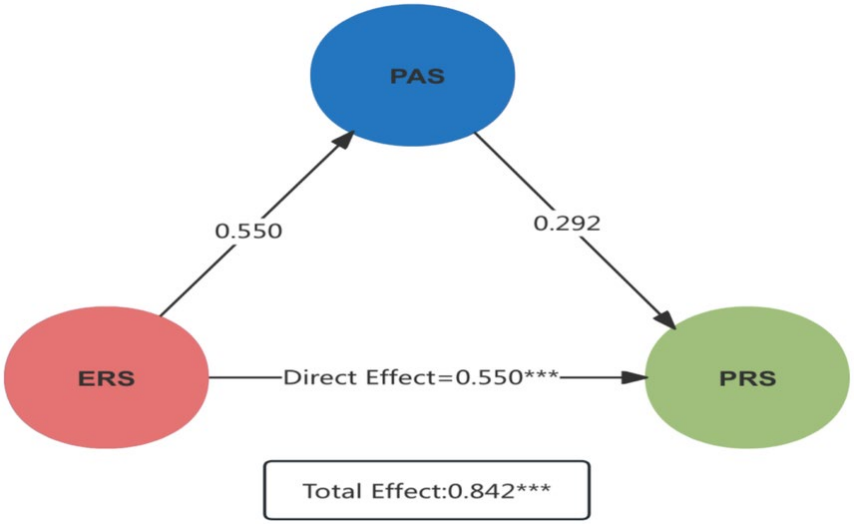
Mediating effect path estimation diagram.

The path analysis results presented in Figure 3 indicate that Emotional Restoration Resources (ERS) have a significant direct effect on Place Attachment (PAS), with a path coefficient of 0.550 (*p* < 0.001). This finding highlights the critical role of emotional restoration resources in shaping individuals’ affective bonds with specific places, thereby enhancing their emotional identification and sense of belonging. The result underscores the central importance of emotional restoration in fostering place-based attachment and psychological identification, which, within the context of rural revitalization, provides empirical support for promoting local participation and community cohesion.

Furthermore, according to Table 8, ERS exerts a significant indirect effect on Perceived Rural Sustainability (PRS) through PAS, with an indirect effect coefficient of 0.292 (*p* < 0.001). This indicates that place attachment serves as a key mediator between emotional restoration resources and sustainable behavior. Specifically, emotional restoration enhances place identity, which subsequently facilitates individuals’ active engagement in green behaviors. This mediation effect confirms the bridging role of place attachment between emotional restoration and sustainable behavior and provides a theoretical foundation for understanding the psychological mechanisms underlying green behavior transformation in rural areas. Overall, the path model depicted in Figure 3 not only elucidates the intrinsic connections among emotional restoration resources, place attachment, and sustainable behavior but also emphasizes the pivotal role of multilevel psychological mechanisms in advancing rural sustainable development. These insights offer a robust theoretical basis for future policy interventions aimed at fostering environmental stewardship and community resilience.

**Table 8:** Mediation path estimates for ERS → PRS.

Effect paths	Effect estimate	Statistical significance
Direct Effect of ERS on PRS	0.55	***
Indirect Effect of ERS on PRS via PAS	0.292	***
Total effect	0.842	***

### 4.3 Measurement reliability, validity, and model fit

Table 9 indicates that all three latent variables demonstrate strong reliability and convergent validity, with composite reliability (CR) values ranging from 0.946 to 0.948 and average variance extracted (AVE) values between 0.854 and 0.858, both exceeding recommended thresholds. The skewness and kurtosis statistics fall within the acceptable range of −1 to +1, supporting the assumption of approximate normality. Although the Shapiro–Wilk test flagged a minor deviation for PRS (*p* = 0.012), such results are not uncommon in larger samples (*N* = 300) and are unlikely to compromise the robustness of SEM estimation. Table 10 shows that all HTMT ratios remain below the conservative criterion of 0.90 (ERS–PAS = 0.675, ERS–PRS = 0.841, PAS–PRS = 0.770), thereby providing further evidence of satisfactory discriminant validity across the constructs.

**Table 9:** Reliability, and convergent validity.

Latent variable	CR	AVE	Skewness	Kurtosis	Shapiro–Wilk p
ERS	0.946	0.854	−0.146	−0.249	*p* = 0.383
PAS	0.948	0.858	−0.131	−0.091	*p* = 0.479
PRS	0.946	0.854	−0.209	−0.367	*p* = 0.012

**Table 10:** HTMT discriminant validity matrix.

Latent variables	ERS	PAS	PRS
ERS	1.000	0.675	0.841
PAS	0.675	1.000	0.770
PRS	0.841	0.770	1.000

Table 11 presents the model fit indices for the three-factor measurement model (ERS, PAS, PRS). The chi-square to degrees of freedom ratio (χ^2^/df = 1.85) was below the commonly accepted cutoff of 3.0, indicating a satisfactory level of parsimony. The incremental fit indices, CFI (0.997) and TLI (0.988), were both above the 0.95 threshold, which can be explained by the clear factor structure and the moderate sample size. More importantly, the RMSEA value (0.053) fell within the acceptable range (≤ 0.08), suggesting that the proposed model provides a reasonable approximation to the population covariance structure. In addition, the SRMR (0.006) was far below the 0.08 benchmark, indicating that the standardized residuals were very small. Overall, these indices confirm that the measurement model fits the data well and is consistent with the underlying theoretical framework.

**Table 11:** Model fit indices.

Index	Value	Recommended threshold
χ^2^	16.612	–
df	9	–
χ^2^/df	1.846	< 3.00 (acceptable)
CFI	0.997	> 0.90 (good), >0.95 (excellent)
TLI	0.988	> 0.90 (good), >0.95 (excellent)
RMSEA	0.053	< 0.08 (acceptable), <0.05 (excellent)
SRMR	0.006	< 0.08 (good)

## 5 Discussion

In the context of rural sustainable development, emotional restoration resources and place identity—central components of psychological mechanisms—have often been underestimated. Yet their contribution to fostering pro-environmental behavior and advancing rural revitalization is substantial. Drawing on systems psychology, this study develops a multilevel framework that integrates emotional restoration, place attachment, and sustainable behavior, thereby illustrating the close interplay between emotional support and place identity in driving green transformation (Hartig et al., 1991; Markevych et al., 2017). The results show that emotional restoration resources strengthen individuals’ sense of place identity, which not only directly encourages pro-environmental actions but also operates as a mediating pathway that promotes rural sustainability. Despite this, current policy frameworks largely overlook such psychological mechanisms, which may reduce the effectiveness of sustainability initiatives. The following discussion elaborates on the broader implications of these findings across several critical dimensions.

### 5.1 Emotional support as an undervalued “soft infrastructure” in rural policy

Emotional restoration resources (ERS), as a form of “soft infrastructure,” are often underestimated in rural policy frameworks. Current revitalization strategies primarily emphasize the development of hard infrastructure—such as transportation, housing, and public facilities—while paying limited attention to emotional and psychological support. The findings of this study demonstrate that emotional restoration strongly facilitates the transformation of green behavior by reinforcing place attachment. This is consistent with evidence showing that exposure to natural environments restores attention, alleviates stress, and reduces rumination, thereby providing affective resources that can be mobilized for prosocial and pro-environmental orientations (Hartig et al., 1991; Korpela et al., 2008; Bratman et al., 2015; Markevych et al., 2017). Empirical analysis further indicates that emotional restoration resources substantially foster place attachment. Yet current rural revitalization policies largely neglect these psychological dimensions, creating a fundamental gap between material development and community well-being. The omission is especially evident in remote areas, where revitalization must encompass not only the upgrading of infrastructure but also the rebuilding of social emotions and local identity. Policies that rely exclusively on economic or technological interventions risk underestimating or delaying their effectiveness if they fail to address the affective bonds that underpin sustainable engagement. Accordingly, future rural revitalization strategies should integrate emotional restoration resources into their frameworks, constructing a dual support system that bridges material improvements with psychological well-being. Such cross-sectoral integration would not only strengthen residents’ place identity and collective cohesion but also enhance the sustainability of policy outcomes by increasing motivation for individual and collective green action.

### 5.2 Psychological mechanism deficits, not technological gaps, undermine green policies

The implementation of green policies often encounters technical and financial challenges; yet the deeper constraint frequently lies in the absence of carefully designed psychological mechanisms. Many existing initiatives overlook the cultivation of emotional identification and psychological support, which results in limited public participation. Prior research demonstrates that efficacy beliefs at the personal, collective, and governmental levels are strong predictors of pro-environmental intentions and policy engagement, providing clear levers for enhancing social acceptance (Jugert et al., 2016; Niles et al., 2024; Sander et al., 2024). The findings of this study reinforce this perspective: emotional restoration resources significantly strengthen place attachment and social trust, thereby facilitating the adoption of green behaviors. Current policy frameworks, however, continue to place disproportionate emphasis on technological and economic interventions while neglecting the cultivation of affective identity and psychological support. This oversight is especially problematic in rural areas of developing countries, where weak cultural identification and emotional alienation reduce local residents’ willingness to participate in sustainability transitions. Rapid social change and the erosion of traditional cultures have further intensified feelings of detachment and resistance, undermining the effectiveness of policy implementation. These findings suggest that the success of green policies depends not only on technical and financial capacity but also on embedding emotional restoration and place attachment into policy design. Integrating these psychological dimensions would increase social acceptance, strengthen enforcement, and enhance long-term sustainability. In doing so, this study highlights a critical gap in current green policy approaches while offering theoretical and practical insights for optimizing future policy frameworks to address socio-psychological barriers in green transitions.

### 5.3 Governance misreads place identity as cultural landscaping

Although this study confirms the mediating role of place identity in fostering green behavior, policy practice often reduces it to superficial “cultural landscape projects.” In many rural revitalization and sustainability programs, local governments emphasize decorative or visual elements of traditional culture while overlooking the deeper psychological mechanisms of place attachment. Place identity, however, extends far beyond symbolic embellishment; it embodies emotions, collective memory, and historical narratives, reflecting residents’ profound affective bonds with their communities. In current policy frameworks, this identity is frequently misinterpreted as cultural symbolism rather than as a genuine response to residents’ emotional needs. Connecting policy interventions to residents’ restorative experiences in everyday contexts—such as favorite places or routine interactions with nearby nature—offers a pathway from aesthetic appreciation to substantive identity and active participation (Korpela et al., 2008; Markevych et al., 2017). Accordingly, rural policy should shift its focus from cultivating “visual identity” to building substantive identity. This requires engaging residents through participatory mechanisms, protecting memory sovereignty, and promoting authentic historical narratives that resonate emotionally. Such strategies are crucial for enabling the genuine transformation and sustainability of green behaviors.

## 6 Conclusion

This study aims to explore the psychological mechanisms underlying emotional restoration resources, place attachment, and green behavior by constructing a multilevel psychological path model based on systems psychology. It reveals the critical roles of emotional support and place identity in rural sustainable development. Using Structural Equation Modeling (SEM) and bootstrap mediation analysis, the study finds that emotional restoration resources significantly predict place attachment, and place attachment serves as an important mediator between emotional restoration resources and green behavior. These results indicate that emotional restoration resources promote the transformation of green behaviors by enhancing place identity. The study fills a theoretical gap regarding the role of emotional restoration and place attachment in green behavior formation, providing new perspectives and theoretical support for psychological interventions in rural revitalization. Compared with previous studies, this research offers several refinements and supplements. Firstly, prior research has mainly focused on the impacts of green policies, technologies, or economic factors on environmental behavior, often neglecting the roles of affective and identity mechanisms. This study posits that emotional restoration resources, as a form of “soft infrastructure,” exert a substantial influence on the generation of green behavior. By emphasizing the dual effects of emotional restoration and place attachment, it deepens the understanding of the relationship between place attachment and green behavior, underscoring the core role of affective identification in driving green behavior. The findings provide new perspectives and theoretical foundations for psychological interventions in rural revitalization, advocating for future policies to emphasize emotional and cultural construction to facilitate green behavior transformation. Notably, this study offers new theoretical explanations for the mechanism by which emotional experiences translate into green behaviors.

Secondly, the study provides a systems psychology framework for the construction of place identity, highlighting that place identity is not merely a cultural symbol but a comprehensive psychological process based on emotions, memory, and social support. This theoretical breakthrough transcends traditional behavioral economic models, offering new pathways to explain the role of place attachment in sustainable behavior. Despite its theoretical and empirical contributions, this study has some limitations. First, the cross-sectional design, although effective for modeling paths between emotional restoration resources, place attachment, and green behavior, lacks the longitudinal data needed to establish causal stability. Future research should employ longitudinal data to verify the long-term effects of emotional restoration and place attachment on green behavior at multiple time points. Second, the sample is geographically concentrated in the eastern and central regions of China, which limits generalizability. Future studies should expand the sample to encompass broader regional and cultural contexts to test the model’s universality. Additionally, factors such as social structure, economic development level, and cultural background in rural communities may moderate the mechanisms of emotional restoration and place attachment; subsequent research could investigate these moderating effects in depth.

Future research directions can be pursued from multiple dimensions. First, integrating larger cross-cultural samples would allow examination of differences in the relationships among emotional restoration resources, place attachment, and green behavior across cultural contexts, enriching the cross-cultural understanding of place identity. Second, the application of big data and intelligent analytic techniques, combining behavioral and psychological state data, can facilitate the development of more precise psychological intervention models, offering personalized pathways to promote green behavior transformation in rural revitalization. In summary, this study not only theoretically enriches the understanding of the relationship between place attachment and green behavior but also provides novel insights for psychological intervention strategies in rural sustainable development. Practically, the construction of emotional restoration resources and place identity offers a psychological support framework for rural green transformation, advancing the transition from “material renewal” to “emotional revival.” Through the application of a systems psychology framework, this research contributes important theoretical support to rural sustainable development and green behavior transformation, bearing significant academic value and policy implications.

## Data Availability

The raw data supporting the conclusions of this article will be made available by the authors, without undue reservation.
